# Oral Microbiota—One Habitat or Diverse Niches? A Pilot Study of Sampling and Identification of Oral Bacterial and Fungal Biota in Patients with Type I Diabetes Mellitus Treated with Insulin Pump

**DOI:** 10.3390/ijerph20032252

**Published:** 2023-01-27

**Authors:** Iwona Gregorczyk-Maga, Mateusz Fiema, Michal Kania, Estera Jachowicz-Matczak, Dorota Romaniszyn, Karolina Gerreth, Tomasz Klupa, Jadwiga Wójkowska-Mach

**Affiliations:** 1 Institute of Dentistry, Faculty of Medicine, Jagiellonian University Medical College, 31-155 Krakow, Poland; 2 Department of Endocrinology, University Hospital, 30-688 Krakow, Poland; 3 Doctoral School of Medicine and Health Sciences, Jagiellonian University Medical College, 31-008 Krakow, Poland; 4 Department of Metabolic Diseases, Center of Advanced Technologies in Diabetes, Faculty of Medicine, Jagiellonian University Medical College, 30-688 Krakow, Poland; 5 Department of Microbiology, Faculty of Medicine, Jagiellonian University Medical College, 31-121 Krakow, Poland; 6 Department of Risk Group Dentistry, Chair of Pediatric Dentistry, Poznan University of Medical Sciences, 60-812 Poznan, Poland

**Keywords:** oral dysbiosis, microbiome, sampling sites, diabetes complications, type 1 diabetes, insulin pump

## Abstract

Objective: The oral microbiota is a very complex and dynamic microbial ecosystem. Alterations of its balance can result in oral and systemic diseases. We aimed to characterize the microbiota in particular niches of the oral cavity in adult type 1 diabetes patients treated with continuous infusion of insulin with insulin pump (IP). In addition, we aimed to determine optimal sites of oral microbiota sampling in studies of large research groups of patients with DM I. Design: In this pilot study, we sampled the buccal and soft palate mucosa, tongue, palatal and buccal dental surfaces and gingival pockets of adult DM I patients treated with IP. Results: In total, 23 patients were recruited. The oral microbiota was dominated by *Streptococus* and *Neisseria*, with a low incidence of cariogenic *S. mutans* and *Lactobacillus*, as well as periodontal pathogens such as *Prevotella*. There were significant differences in overall CFU counts of all strains, Gram-positive, *Staphylococci*, *Streptococci* and *S. oralis* strains between mucosal and dental surface sites. The overall CFU counts of all strains and Gram-positive strains were higher in dental sites vs. mucosal sites (both *p* < 0.001). CFU counts of S. oralis were significantly higher in dental sites vs. gingival pocket sites (*p* = 0.013). Candida species were rare. The mucosal sites on the buccae presented lower diversity and bacterial counts. Conclusions: In the study group of adult DM I patients treated with IP, the microbiota in particular niches of the oral cavity was significantly different. Three distinct and optimally appropriate sampling sites for oral microflora were identified: buccal and palatal mucosa, dental surface and gingival pockets. The results of this study may be the basis for further studies of large groups of patients with DM I.

## 1. Introduction

The oral microbiota is one of the largest and most diverse microbial ecosystems, and plays an important role in maintaining human health [1]. The oral microbiota is distinctively different from the ecosystems of the remaining digestive tract sections [2], with exceptional microbiological diversity. Alterations of the oral microbiota balance can result in both oral (e.g., periodontal diseases, caries) and systemic (e.g., cardiovascular disease, pre-term childbirth) issues [1].

Type 1 diabetes mellitus (DM I) is a chronic condition in which the pancreas produces little or no insulin, leading to hyperglycemia. Continuous subcutaneous insulin infusion with insulin pump (IP) is a modern method of insulin administration, with evidence for its superiority to traditional multiple daily injections in terms of metabolic control, lower risk of hypoglycemic events and better quality of life, especially when used with continuous glucose monitoring systems [3]. Insulin pumps have been available in Poland since the mid 1990s. When good metabolic control is not reached, diabetic complications can occur, including neuropathy, nephropathy and retinopathy, as a result of microangiopathy, as well as macro-angiopathic cardiovascular disease [4]. A bidirectional relationship between oral health and DM I has been suggested as a predisposing factor to oral infections, which, in turn, exacerbates the progression of systemic disease [5]).

Decreased secretion of aberrant saliva (of lower pH and more concentrated) can lead to xerostomia, increased caries risk and higher susceptibility to *Candida* sp. infections. The formation of advanced glycosylation end products (AGEs) and their deposition in tissues leads to microvascular damage and vascular dysfunction due to hyperglycemia. This causes immunological dysregulation, ineffective wound healing, a decrease in the regenerative potential of the mucosa, gingival resorption and periodontal diseases [6,7,8,9].

Two large databases, the Human Microbiome Project (HMP) and Human Oral Microbiome Database (HOMD), were developed through recent extensive research. The HMP encompasses microbiome data from the oral and nasal cavities, vagina, gut and skin. Data in the HOMD incorporate oral microbiota composition [10].

The oral cavity is a complex and diverse microbial ecosystem, and has to be divided into various niches colonized by distinct microorganisms [1,11]. These individual habitats can be sampled by swabbing or brushing, testing saliva or oral rinses [12].

Previous reports of oral microbiota in adult DM I are scarce, focusing rather on children or adolescents, or patients with poor oral health, with caries or periodontal disease. DM I patients with worse metabolic control of diabetes present with aberrations in the oral microbiome and its progressive dysbiosis [13,14]. In contrast, DM I patients without complications and with good metabolic control may not show oral pathology [15], but greater abundance of *Streptococcus* spp., *Actinomyces* spp. and *Rothia* spp. compared to otherwise healthy controls was observed [15]. To date, no attempts have been made to characterize the microbiota of individual niches in the oral cavity in DM I, with studies analyzing mainly the saliva or swabs from single sites.

The aim of this pilot study was to characterize the microbiota in particular niches of the oral cavity in adult DM I patients treated with IP. In addition, we aimed to determine optimal sites of oral microbiota sampling in studies of large research groups of patients with DM I.

## 2. Materials and Methods

### 2.1. Study Design and Participants

This pilot study consecutively recruited 23 adult patients with DM I treated with IP from the Outpatient Clinic of the Department of Metabolic Diseases and Diabetology of the University Hospital in Krakow, an academic referral center for diabetes in southeastern Poland. Patients who met inclusion criteria were invited to participate in the study. After acquiring written consent, the date of sampling was set, and the patients were instructed on how to prepare for the study procedures. The inclusion criteria were: patient 18–35 years old; DM I diagnosed at least 1 year before recruitment; treatment with IP for at least 6 months; informed consent to participate in the study. The exclusion criteria were: pregnancy or breastfeeding; and comorbidities such as metabolic syndrome, cardiovascular disease, cancer, severe liver failure or kidney failure. The diagnosis of DM I was confirmed based on the Diabetes Poland criteria [16]. Data on age, gender, fasting glucose levels on the day of sampling, glycated hemoglobin (HbA1c%) and DM I treatment were extracted from medical records. HbA1c% was measured using high-performance liquid chromatography (HPLC) or an enzymatic method within 1 month prior to sampling.

The preparation for collecting microbiological samples from the oral cavity involved refraining from brushing the teeth with triclosan toothpastes and rinsing with chlorhexidine for 48 h preceding the visit. On the day of examination, the patients refrained from brushing their teeth and drinking, eating or smoking for 1 h before microbiological samples were collected. One study subject was excluded from analysis due to failing to comply with the study standards (after sampling, the subject admitted to brushing their teeth and eating breakfast within 1 h preceding the study visit).

### 2.2. Oral Cavity Sampling Methods

An oral assessment was performed prior to sample collection. The general condition of the oral cavity was assessed using the Oral Health Assessment Tool (OHAT) [17].

Six oral habitats were sampled: buccal (marked A) and soft palate (B) mucosa, the tongue (C), palatal (Da) and buccal (Db) dental surface, and the gingival pocket (E). The niches of each subject were sampled once. Samples were taken by an experienced dentist on an operating chair equipped with an operating light. Specimens from the posterior part of the dorsum of the tongue and soft palate were collected using an ESwab™ [18]. The ESwab combines a COPAN-invented flocked swab with 1 mL of Liquid Amies in a plastic screw cap tube. Dental plaque was collected from the buccal and palatal dental surface sides using a Tooth Cleanic KerrHawe (Dental Supplies—Dental Products|KerrDental.Com, Kloten, Switzerland [19]). After collection, the brush was placed in 1 mL of Liquid Amies in a plastic screw cap tube. A periodontal probe was used to examine the depth of the gingival pocket. Afterwards, two pieces of PerioPaper Strips [20], which are designed to absorb 0–1.2 micro-liters of fluid, were used to collect gingival crevicular fluid (GCF) samples. The strips were placed in the deepest part (1–2 mm) of the gingival pocket for 30–45 s till their surface was soaked. To minimize the risk of pre-analytical errors during sample collection, sterile gauze was used to remove excess saliva from the mucosae and dry the dental surfaces, preventing salivary contamination of the GCF.

### 2.3. Microbiological Cultures

The collected samples were immediately delivered to the microbiological laboratory. The samples were then inoculated using the dilution method (dilutions −1 to −6) or qualitative culture method (swabs only) on the following media: McConkey (Graso, Biotech Starogard Gdański, Poland [21]), Columbia (Lab-Agar, Biomaxima, Lublin, Poland [22]), Scheadler (Scheadler-Agra, Biomaxima, Lublin, Poland [22]), Bile Esculine Azide (Lab-Agar, Biomaxima, Lublin, Poland), MRS Agar (Oxoid, Brno, Czech Republic [23]) or Sabouraud Agar (Biomaxima, Lublin, Poland [22]). Media were aerobically incubated at 37 °C (McConkey, Columbia, Bile Esculine Azide and Sabouraud) for 24 h, or anaerobically at 37 °C (M.R.S and Scheadler) for 48 h. After incubation, the phenotypically grown colonies were counted and reported, with the results being presented as colony-forming units (CFU) per mL (CFU/mL). After isolation, the microorganisms were identified through MALDI TOF MS mass spectrometry (MALDI Biotyper, Bruker [24]).

### 2.4. Statistical Analysis

PS Imago Pro ver. 6.0, Statistica ver. 13 and PQStat ver. 1.8.2 were used for all statistical analysis. The normality of the continuous variable distribution was assessed using the Shapiro–Wilk test. Differences between groups were analyzed with Student’s *t* test or nonparametric tests (Mann–Whitney U test, Kruskal–Wallis ANOVA), when appropriate. Paired data were analyzed using the Wilcoxon test, Friedman’s ANOVA and Skellings–Mack ANOVA, along with appropriate post hoc tests. Continuous variables were presented as arithmetic means ($\bar{x}$) ± standard deviations (SD) or as the median with interquartile range (IQR) when the data were not normally distributed. The distribution of categorical variables was described as counts and percentages. Statistical testing was completed to compare categorical variables using an independent sample chi-squared test or Fisher’s exact test when appropriate, and dependent samples using McNemar’s test and Cochrane’s Q ANOVA. Statistical significance was set at *p* < 0.05. The Bonferroni method was used to correct for multiple comparisons.

The analyzed variables were: age, gender, fasting glucose levels on the day of sampling, glycated hemoglobin (HbA1c%), OHAT score, number of genera and species in respective sites and bacterial and fungal CFU counts.

**Figure 1:**
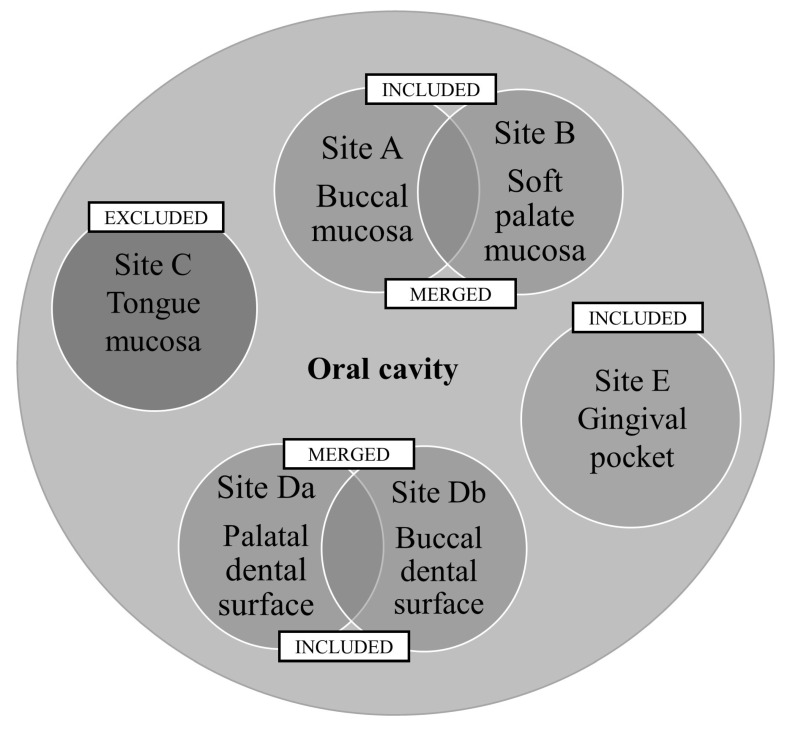
Flowchart of sites selected for analysis.

In the first analysis, we compared mean CFU counts between all sites (A–E). Since the total number of CFUs of all strains on the back of the tongue (site C) was the highest and significantly different from the other sites, potentially distorting the results, we decided to exclude it from further analyses. In the second, we performed an analysis of sites A–E with site C excluded. Finally, based on the acquired results and previous studies suggesting treating mucosal (both palatal and buccal) and dental (buccal and lingual) surfaces as two separate habitats [12], we merged the sites A and B, and Da and Db, and compared them with site E (Figure 1).

### 2.5. Ethics Statement

This study was approved by the Jagiellonian University Bioethics Committee, decision number 1072.61.20.10.2021. Written informed consent was obtained from each subject prior to participation.

## 3. Results

Data from 22 subjects with DM I treated with a continuous subcutaneous insulin infusion were analyzed. The mean age of the sample was 27.05 ± 5.95 years. The sample was predominantly male (*n* = 13; 59.1%). The mean Hba1c% was 6.97 ± 0.95%. The mean fasting glycemia was 116.24 ± 38.29 mg/dL. The condition of the oral cavity according to the OHAT scale in 19 participants was normal (total score 0/16—no change). Three participants had benign changes (total score 1/16, due to dental plaque accumulation). All participants had gingival pocket depths of 1–2 mm, which is considered healthy. None of the participants had a history of any dental procedure in the 6-month period preceding the study procedures. The basic characteristics of the study sample are presented in Table 1.

**Figure 2:**
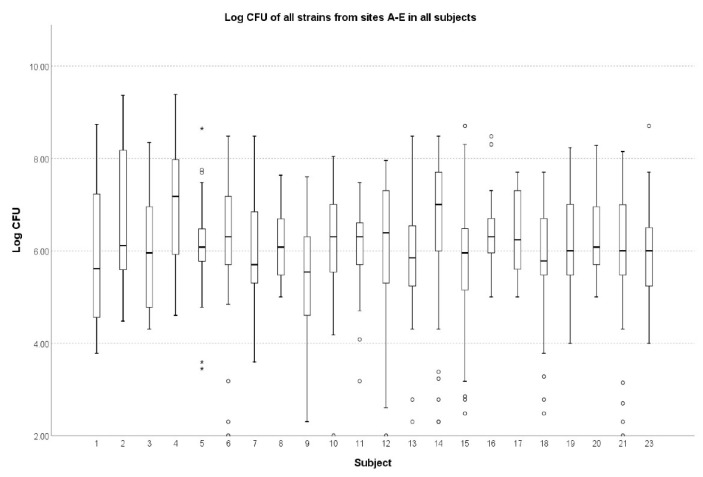
Log CFU of all bacterial and fungal strains from all sites A–E. * extreme values, ° outliers.

There were no significant differences in the number of microbial genera and species from sites A to E (*p* = 0.459). Except for one significant difference between subjects 4 and 9 (median, IQR: 15 × 10^7^, 7 × 10^7^–93 × 10^6^ vs. 35 × 10^14^, 35 × 10^13^–275× 10^4^; *p* = 0.045), the patients did not differ between the sites for the overall CFU counts (Figure 2).

**Figure 3:**
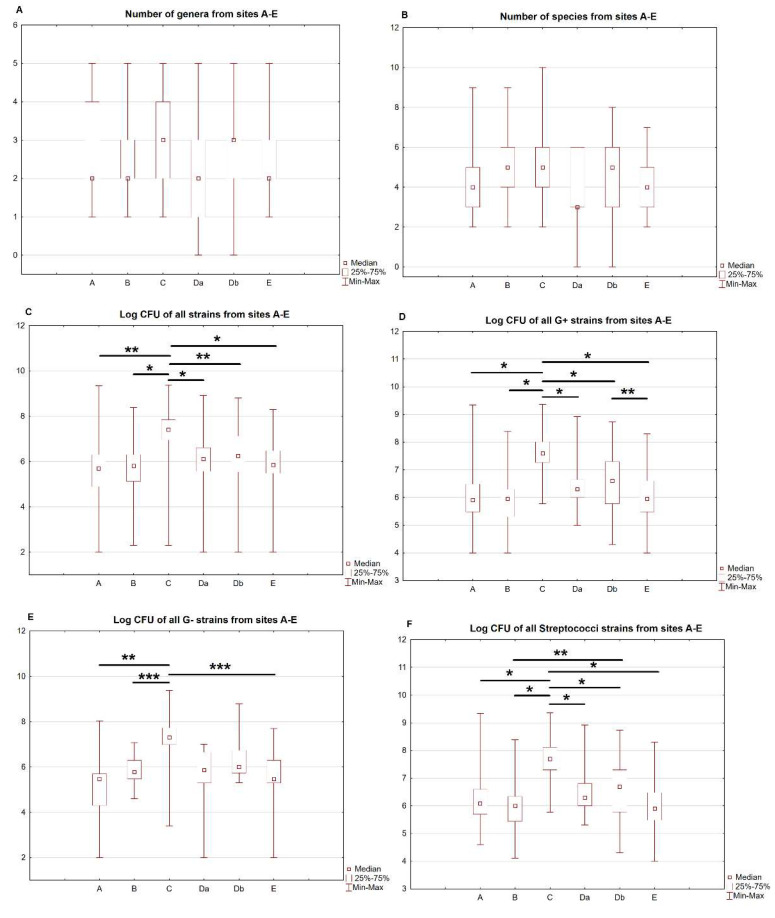
(**A**–**F**) Comparisons of sites A–E. Number of species (**A**) and genera (**B**) from sites A–E. Log CFU of all strains (overall, **C**), G+ (**D**), G− (**E**) and Streptococci strains (**F**) from sites A–E. * *p* < 0.001; ** *p* < 0.01; *** *p* < 0.05.

In the first prespecified analysis, the number of genera and species, and CFU counts between all sites for all identified strains and with division into G-positive, G-negative, streptococci species (all identified strains) and staphylococci species (all identified strains) were compared. There were no significant differences in the number of genera and species between the sites. The overall CFU counts of all strains in site C were the highest and significantly differed from the remaining sites (*p* < 0.001 for all comparisons). The detailed results are presented in Table 2. The comparison of the different sites is presented in Figure 3A–F.

In the second step, site C was excluded from the analysis. Then, CFU counts between all remaining sites for all identified strains and with division into G-positive, G-negative, streptococci (all identified strains) and staphylococci strains (all identified strains) were compared. There were significant differences in the overall CFU counts of all strains, Gram-positive, streptococci and staphylococci strains between the sites. Post hoc analysis showed that the only significant differences were limited to comparisons of mucosal sites and dental surfaces. Overall CFU counts of all strains were higher in site Db vs. A (*p* = 0.011) and site Db vs. B (*p* = 0.001), and CFU counts of streptococci were higher in site Db vs. B (*p* = 0.007). These results are presented in Table 2.

Finally, sites A and B were merged into category A + B (mucosal sites), and Da and Db into Da + Db (dental surfaces), and compared with site E. There were significant differences in the overall CFU counts of all strains, Gram-positive, staphylococci, streptococci and *S. oralis* strains between the sites. Post hoc analysis showed significant differences in the merged mucosal and dental surfaces. The overall CFU counts of all strains and Gram-positive strains were higher in sites Da + Db vs. A + B (both *p* < 0.001). CFU counts of *S. oralis* were significantly higher in Da + Db vs. E (*p* = 0.013). There were also some borderline significant results; CFU counts of streptococci (*p* = 0.071), *S. oralis* (*p* = 0.065) and staphyloccoci (*p* = 0.083) strains tended to be higher in Da + Db vs. A + B. Cariogenic *S. mutans* was identified in only three samples from three study subjects, and was found in sites Da (one sample, CFU 3 × 10^7^) and Db (two samples, CFU 6 × 10^5^ and 2 × 10^6^). *Candida* was identified in all sites except for site E. *C. albicans* dominated the samples, with only four *C. dubliniensis* strains being identified. The only significant difference was found in the overall CFU counts of all *Candida* species between the merged sites A + B and Da + Db, with significantly higher counts of *Candida* on the dental surfaces (*p* = 0.015). In one subject, one strain of *Geotrichum* spp. was identified.

**Figure 4:**
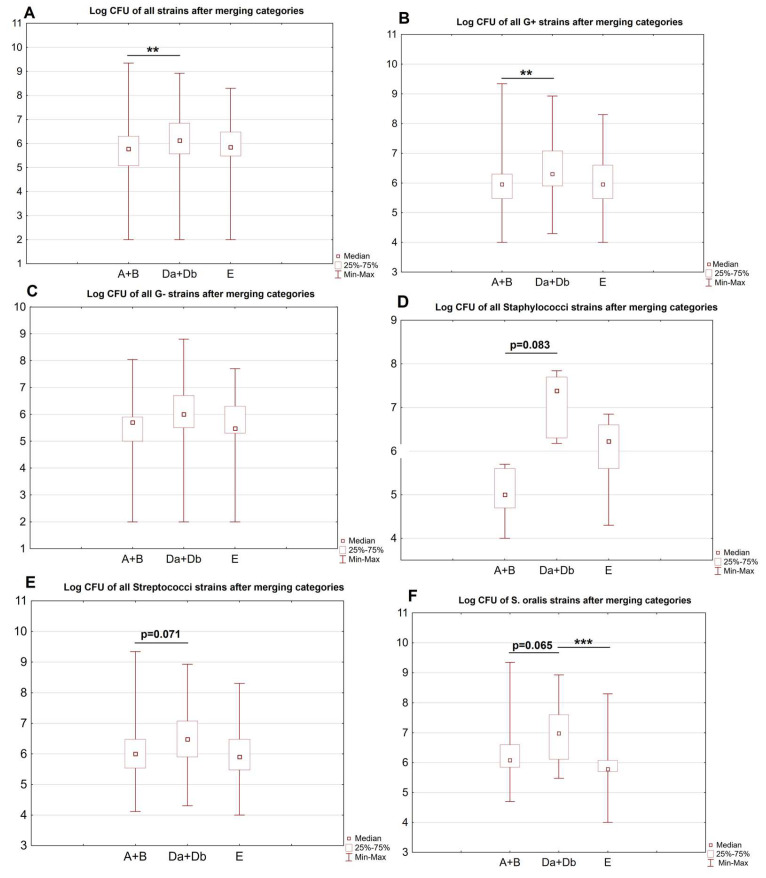
(**A**–**F**) Comparisons of sites A + B, Da + Db and E. Log CFU of all strains (overall, A), G+ (B), G− (**C**), *Staphylococci* (**D**), *Streptococci* (**E**) and *S. oralis* (**F**) strains after merging categories. Selected borderline significant differences are presented. ** *p* < 0.01; *** *p* < 0.05.

The results are presented in Table 3. The comparison of merged categories is presented in Figure 4. The detailed information concerning all identified strains of bacteria and fungi is presented in Supplementary Material Table S1.

## 4. Discussion

Patients with DM I are at risk of developing numerous oral-related pathologies, such as mucosal disorders, caries, and periodontal disease [6,7,8,25]. The microvascular damage and vascular dysfunction evident in patients with DM I result in higher susceptibility to oral lesions, impaired wound healing, abnormal bleeding during dental procedures and a higher prevalence of mucosal disorders such as candidiasis [7,26,27]. Most studies to date have investigated patients with DM II, but this sample differs from our patients, who were all diagnosed with DM I. Previous reports of oral microbiota in adults with DM I are scarce, focusing rather on children or adolescents, or patients with poor oral health and those with caries or periodontal diseases [26,28,29]. Our study sample included homogenous, relatively young patients with DM I, without diabetic complications or excess cardiovascular burden. The sample was already described in another study [30], showing exceptional metabolic control. Our study population had good oral health, with no significant signs of periodontal pathology.

In this report, for the first time to the best of our knowledge, we thoroughly characterized the oral bacterio- and mycobiota with traditional culture-based methods, with MALDI-TOF species identification in adult patients with DM I, treated with IP and achieving satisfactory metabolic control. In these patients, the characteristics of the oral microbiota were comparable to previously reported data from otherwise healthy controls. Despite the health and financial burden imposed by DM I, a portion of IP-treated patients with good metabolic control may not present significant abnormalities in oral health and microbiota, confirming our initial hypothesis.

Most studies have mainly analyzed saliva or swabs obtained from single sites, with a paucity of available data regarding the diversity of microbiota in individual niches in the oral cavity of patients with DM I. In this pilot study, we also confirmed our hypothesis that the oral cavity consists of several separate microbiologically distinct habitats. We defined the three most appropriate sites for sampling the oral microbiota for future studies: The buccal and palatal mucosa, dental surfaces and gingival pockets.

In the general population, the oral microflora consists mainly of Firmicutes (*Streptococcus*, *Veillonella*, *Granulicatella*), Proteobacteria (*Neisseria*, *Haemophilus*), Actinobacteria (*Corynebacterium*, *Rothia*, *Actinomyces*), Bacteroidetes (*Prevotella*, Capnocytophaga, *Porphyromonas*) and Fusobacteria (*Fusobacterium*) [3,11,31,32]. In our sample, the oral microbiota was dominated by *Streptococus* and *Neisseria* taxa, with a low incidence of cariogenic *S. mutans* and *Lactobacillus*, as well as periodontal pathogens such as Porphyromonas, Prevotella and *Treponema*.

Our results were similar to reports of previous studies including patients with DM I. In one study of adults with DM I without complications and with good metabolic control (HbA1c < 10%), the participants had a greater abundance of *Streptococcus* spp., *Actinomyces* spp. and *Rothia* spp. than otherwise healthy controls. However, DM I was not associated with oral pathology. Similarly, there were no significant correlations between oral microbiota and glycemic control [15]. Another study, which included children with DM I, reported *Streptococcus* as one of the largest groups of isolated microorganisms [33]. Finally, a recent study of children with DM I that used similar traditional methods of bacteria culture and identification reported significantly higher numbers of bacteria from the *Streptococcus* genus in the group of children with well-controlled DM I than otherwise healthy controls [26]. Another study reported higher abundance of *S. mitis* and lower abundance of *S. salivarius* in DM I individuals, linking this with inter-microbial competition [34]. In contrast, patients with poor glycemic control exhibited worse oral health status with higher frequency of caries and gingivitis [35]. Maintaining metabolic control may partially ameliorate oral microbiota dysbiosis in DM I patients [36]. Considering the plaque microbiota, worse glycemic control was associated with more complexity and richness, with increasing HbA1c levels [13,37].

*Candida* species were also rare in our samples, dominated by common commensal *C. albicans* strains. Few studies reported the relationships of DM I with mycobiota. Fungi, including selected *Candida* species, *Aspergillus*, *Fusarium* and *Saccharomyces* species, have been associated with healthy oral microbiota [38]. However, some *Candida* species have been identified as a risk factor for oral pathology, such as periodontal disease [39,40]. In contrast to previous studies, we showed a higher load of *Candida* on the oral mucosa in patients with DM I [9]. Interestingly, one identified strain of *Geotrichum* was previously reported to be used as a cheesemaking culture [41], which confirms, on the one hand, the importance of patient preparation for the examination in such a study and, on the other hand, how variable and dependent on the environment or patient behavior the oral microbiota is.

The data concerning adults with DM I treated with IP are extremely limited, thus making our findings noteworthy. Patients in this study had no history of oral cavity pathology and showed good oral health, which, however, was not the intention of the authors—the good or bad condition of the oral cavity was not a qualification criterion. In addition, the fact that our subjects had exceptional metabolic control resulted in the healthy characteristics of the oral microbiota.

The oral cavity cannot be treated as one large, homogenous microbial ecosystem. It is extremely complex and diverse, and has to be divided into various niches colonized by distinct microorganisms [1,11]. Shedding (mucosae) and non-shedding (dental surfaces) surfaces form two main, compositionally separate communities [12]. Dental sites can be divided into supra- and sub-gingival (gingival pocket, gingival crevicular fluid). Epithelial surfaces are covered with non-keratinized covering mucosa (oral floor, buccae, labiae, soft palate), keratinized masticatory mucosa (gingiva and hard palate) and papillary mucosa (tongue dorsum) [42]. Finally, saliva includes bacteria originating from various niches, bearing some resemblance to the tongue surface [12,42].

In our study, we compared multiple niches and sample types to facilitate the selection of appropriate ones for investigating the oral microbiota of DM I. The dorsal surface (mucosal swab) of the tongue was the richest in microorganisms, and showed shared biodiversity with the remaining oral sites. These findings are in keeping with previous studies that reported a significant abundance of bacteria accumulated from various oral niches [12,43], thus making it is less useful for site-specific research. The mucosal sites on the buccae (mucosal swab) presented lower taxonomical diversity and lower bacterial counts. Some studies reported that mucosal swabs of the buccae and palate, though easily obtainable, show low bacterial diversity and are usually contaminated by microorganisms from other surfaces such as the tongue or teeth [12,44]. Finally, we identified the dental surfaces (by brush) as distinctive to mucosal sites, with higher bacterial counts. Supragingival dental plaque, formed by the biofilm covering the dental surfaces, represents a specific dental surface, and allows distinction between caries lesions and healthy surfaces. However, it requires a trained professional and clinical setting for sampling [12,45,46]. The gingival pockets did not show any significant quantitative differences from the remaining sites. We believe that this may have resulted from the good oral health of our subjects, who had no history of periodontal disease, and the fact that some subgingival-specific strains can be undetected with traditional identification methods [2]. Interestingly, one study showed buccal samples, as compared to subgingival plaque, provided better distinction between patients with periodontitis and otherwise healthy controls [47]. Nevertheless, subgingival plaque is highly relevant to oral health, but requires professionals to perform the procedure [12].

As shown in previous studies, each of these habitats can be sampled with different method and equipment, including swabbing and brushing with dedicated equipment, testing saliva or oral rinses [12]. Each method has its own advantages and disadvantages, such as the possibility of being performed by non-professionals or no requirements for special equipment. However, these advantages come with a cost of imprecision [12]. Currently, the techniques based on next-generation sequencing of the 16 rRNA remain the gold standard for diagnostics in microbiome research. The conventional culture-dependent techniques can still provide useful information on the bacterial diversity on the subspecies level, and identify non-bacterial organisms, such as *Candida* [2,12].

To sum up, we identified three distinct and most appropriate sites for sampling the oral microbiota in DM I patients: buccal and palatal mucosa, dental surfaces and gingival pockets. This finding may facilitate the choice of adequate methods for similar future studies. Nevertheless, the aim of an individual study and its design should inform the choice of methods and sites used to sample the oral cavity. To investigate gross oral microbiota, especially in larger cohorts, sampling saliva can be appropriate [12], but may fail to discriminate between clinically relevant differences [48]. When researching total oral biodiversity, the optimal approach combines multiple samples from different niches [12]. In studies of site-specific pathologies, such as periodontal diseases or caries, site-specific sampling is recommended [12]. Swabbing or brushing are considered the most reliable methods [12], while saliva and oral rinses, though non-invasive and easy to obtain, are merely a proxy for oral microbiota, not representing any specific niche [49,50].

Our study has some limitations. Since this was a pilot study aimed at the initial assessment of the oral microbiota and the selection of optimal niches for the collection of microbiological samples, it was decided to include only patients with DM I in the first stage and compare them narratively with healthy controls described in previous studies (Verma et al., 2018; Zaura et al., 2009). We used the basic methods of identifying oral microorganisms as a starting point for further, more advanced diagnostic methods. The assessment of the microbiota will be extended with metagenomic analyses. Funding for this study was granted by the Polish Ministry of Science and Higher Education (grant number NdS/545131/2022/202). The subjects were all in relatively good health, with good metabolic control, no diabetic complications and no oral pathologies, so our findings are only applicable to a similar subpopulation of DM I patients. Another limitation was associated with the nature of microbial count data. Since the data were, in most cases, non-normal, paired and unbalanced, they required specific methods of statistical analysis that are usually more conservative than tests for parametric or balanced data. Therefore, some statistical power was lost; however, importantly, the type I error probability was decreased.

## 5. Conclusions

The oral cavity cannot be treated as one large, homogenous microbial ecosystem. In the study group of adult patients with type 1 diabetes treated with continuous infusion of insulin with insulin pump, the microbiota in particular niches of the oral cavity was significantly different. Three distinct and optimally appropriate sampling sites for oral microbiota have been identified: buccal and palatal mucosa, dental surfaces and gingival pockets. The results of this study may be the basis for further studies of large groups of patients with DM I.

## Figures and Tables

**Table 1:** Characteristics of the study population.

Clinical Feature	Available Data, *N* (%)	Mean (SD), Median (Q1–Q3) or Number (%)	*p*-Value
Age	22 (100%)	27.05 (5.95) 26.5 (22–29.25)	-
Gender [male]	22 (100%)	13 (59.1%)	-
Weight [kg]	15 (68.2%)	71.78 (17.75) 66.0 (57–90.0)	-
HbA1c [%] $	22(100%)	6.97 (0.95) 6.85 (6.3–7.35	
Fasting glycemia [mg/dL]	21 (95.5%)	116.24 (38.29) 112.0 (93.0–131.5)	-
OHAT [score]	22 (100%)	0/16 (19, 86.4%) 1/16 (3, 13.6%)	-
Microbial Counts			
Number of genera	22 (100%)		0.459
A		2 (1.75–4)	
B		2 (2–3)	
C		3 (2–4)	
Da		2 (1–3)	
Db		3 (2–3)	
E		2 (2–3)	
Number of species	22 (100%)		0.459
A		4 (3–5)	
B		5 (4–6)	
C		5 (3.75–6.25)	
Da		3 (2.75–6)	
Db		5 (3–6)	
E		4 (3–5)	
Mean CFU [CFU/mL]	22 (100%)		0.018 #
All sites A–E		3.88 × 10^7^ ± 1.88 × 10^8^ 1.20 × 10^6^ (3.00× 10^5^ –1.00 × 10^7^)	

OHAT—Oral Health Assessment Tool, CFU—colony-forming unit; $—HbA1c measured using high-performance liquid chromatography or enzymatic method; buccal (A) and soft palate (B) mucosa, the tongue (C), palatal (Da) and buccal (Db) dental surface, gingival pocket (E); #—in post hoc analysis, significant difference for comparison between subjects 4 and 9 (*p* = 0.045).

**Table 2:** Characteristics of sites A–E.

Site	A	B	C	Da	Db	E	*p*-Value(A–E)	*p*-Value(A–E, exl. C)
No. of genera	2 (1.75–4)	2 (2–3)	3 (2–4)	2 (1–3)	3 (2–3)	2 (2–3)	0.299	-
No. of species	4 (3–5)	5 (4–6)	5 (3.75–6.25)	3 (2.75–6)	5 (3–6)	4 (3–5)	0.124	-
No. of samples [*N*], CFU [CFU/mL]								
Overall &	100 4.34 × 10^7^ (2.40 × 10^8^) 5.00 × 10^5^ (7.48 × 10^4^–2.00 × 10^6^)	112 8.25 × 10^6^ (3.56 × 10^7^) 6.50 × 10^5^ (1.28 × 10^5^–2.00 × 10^6^)	114 1.05 × 10^8^ (3.17 × 10^8^) 2.50 × 10^7^ (8.75 × 10^6^–7.00 × 10^7^)	80 2.08 × 10^7^ (1.09 × 10^8^) 1.30 × 10^6^ (3.55 × 10^5^–4.00 × 10^7^)	96 2.57 × 10^7^ (8.93 × 10^7^) 1.75 × 10^6^ (3.25 × 10^5^–1.43 × 10^7^)	89 7.17 × 10^6^ (2.43 × 10^7^) 7.00 × 10^5^ (3.00 × 10^5^–3.00 × 10^6^)	<0.001 #	0.001 *
Gram-positive &	72 5.85 × 10^7^ (2.82 × 10^8^) 8.00 × 10^5^ (3.00 × 10^5^–3.00 × 10^6^)	82 1.09 × 10^7^ (4.13 × 10^7^) 9.00 × 10^5^ (2.00 × 10^5^–2.00 × 10^6^)	76 1.08 × 10^8^ (2.76 × 10^8^) 3.90 × 10^7^ (1.83 × 10^7^–1.06 × 10^8^)	52 3.12 × 10^7^ (1.34 × 10^8^) 2.00 × 10^6^ (1.00 × 10^6^–4.75 × 10^6^)	63 2.73 × 10^7^ (7.77 × 10^7^) 4.00 × 10^6^ (6.00 × 10^5^–2.00 × 10^7^)	67 8.20 × 10^6^ (2.73 × 10^7^) 9.00 × 10^5^ (3.00 × 10^5^–4.00 × 10^6^)	<0.001 $	0.001 φ
Staphylococci &	4 2.03 × 10^5^ (1.75 × 10^5^) 1.80 × 10^5^ (5.50 × 10^4^ –3.50 × 10^5^)	5 2.06 × 10^5^ (2.28 × 10^5^) 1.00 × 10^5^ (2.00 × 10^4^–4.00 × 10^5^)	7 5.00 × 10^7^ (7.76 × 10^7^) 2.00 × 10^7^ (1.00 × 10^6^–6.00 × 10^7^)	2 1.75 × 10^6^ (3.54 × 10^5^) 1.75 × 10^6^ (1.50 × 10^6^–2.00 × 10^6^)	4 4.25 × 10^7^ (2.25 × 10^7^) 4.10 × 10^7^ (2.50 × 10^7^–6.00 × 10^7^)	6 2.47 × 10^6^ (2.63 × 10^6^) 1.70 × 10^6^ (4.00 × 10^5^–4.00 × 10^6^)	0.005 φ	0.03 φ
Streptococci &	61 6.90 × 10^7^ (3.05 × 10^8^) 1.20 × 10^6^ (5.00 × 10^5^ –4.00 × 10^6^)	67 1.32 × 10^7^ (4.54 × 10^7^) 1.00 × 10^6^ (2.80 × 10^5^–2.20 × 10^6^)	58 1.27 × 10^8^ (3.12 × 10^8^) 4.95 × 10^7^ (2.00 × 10^7^–1.30 × 10^8^)	44 3.66 × 10^7^ (1.46 × 10^8^) 2.00 × 10^6^ (1.00 × 10^6^–6.50 × 10^6^)	51 2.99 × 10^7^ (1.46 × 10^8^) 5.00 × 10^6^ (6.0 × 10^5^–2.00 × 10^7^)	51 8.66 × 10^6^ (3.06 × 10^7^) 8.00 × 10^5^ (3.00 × 10^5^–3.00 × 10^6^)	<0.001 ψ	0.001 §
Gram-negative &	18 6.64 × 10^6^ (2.58 × 10^7^) 3.00 × 10^5^ (1.88 × 10^4^–5.50 × 10^5^)	19 1.62 × 10^6^ (2.87 × 10^6^) 6.00 × 10^5^ (3.00 × 10^5^–2.00 × 10^6^)	19 5.01 × 10^7^ (5.31 × 10^8^) 2.00 × 10^7^ (1.00 × 10^7^–4.00 × 10^7^)	12 2.59 × 10^6^ (3.42 × 10^6^) 7.50 × 10^5^ (2.00 × 10^5^–4.75 × 10^6^)	20 3.71 × 10^7^ (1.40 × 10^8^) 1.00 × 10^6^ (5.25 × 10^5^–5.75 × 10^6^)	19 4.55 × 10^6^ (1.17 × 10^7^) 3.00 × 10^5^ (2.00 × 10^5^–2.00 × 10^6^)	<0.001%	0.054
*Candida* spp.	5 9.00 × 10^2^ (8.33 × 10^2^) 5.00 × 10^2^ (2.00 × 10^2^–1.80 × 10^3^)	4 4.50 × 10^2^ (2.38 × 10^2^) 4.50 × 10^2^ (2.25 × 10^2^–6.75 × 10^2^)	9 1.52 × 10^3^ (1.84 × 10^3^) 1.20 × 10^3^ (2.00 × 10^3^–1.95 × 10^3^)	9 1.64 × 10^5^ (4.28 × 10^5^) 4200 (2.70 × 10^3^–7.50 × 10^4^)	9 2.86 × 10^4^ (7.2 × 10^4^) 2.80 × 10^3^ (4.50 × 10^2^–1.38 × 10^4^)	0	0.22	0.23

Data are presented as mean (SD), median (Q1–Q3) or number. Buccal (A) and soft palate (B) mucosa, the tongue (C), palatal (Da) and buccal (Db) dental surface, gingival pocket (E). &—all identified strains. #—significant difference C vs. A. B. Da. Db. E at *p* < 0.001. *p* < 0.001. *p* = 0.001. *p* = 0.026 and *p* < 0.001, respectively. $—significant difference C vs. A. B. Da. Db. E at *p* < 0.001 in all comparisons and E vs. B. Db at *p* = 0.001 and *p* = 0.003, respectively. %—significant difference C vs. A. B. E at *p* = 0.037; *p* = 0.005 and *p* = 0.038, respectively. ψ—significant difference C vs. A. B. Da. Db. E at *p* < 0.001 in all comparisons and B vs. Db at *p* = 0.009. *—significant difference A vs. Db. *p* = 0.011; B vs. Db. *p* = 0.001. φ—no significant differences in the post hoc analysis. §—significant difference B vs. Db. *p* = 0.007.

**Table 3:** Characteristics of sites after merging categories A + B and Da + Db.

Site	A + B	Da + Db	E	*p*-Value
No. of cultures [*N*], CFU counts [CFU/mL]				
Overall #	212 2.48 × 10^7^ (1.67 × 10^8^) 6.00 × 10^5^ (1.20 × 10^5^–2.00 × 10^6^)	176 2.34 × 10^7^ (9.85 × 10^7^) 1.35 × 10^6^ (3.70 × 10^5^–7.00 × 10^6^)	89 7.17 × 10^6^ (2.43 × 10^7^) 7.00 × 10^5^ (3.00 × 10^5^–3.00 × 10^6^	0.006 $
Gram-positive #	154 3.32 × 10^7^ (1.96 × 10^8^) 9.00 × 10^5^ (3.00 × 10^5^–2.00 × 10^6^)	115 2.91 × 10^7^ (1.07 × 10^8^) 2.00 × 10^6^ (8.00 × 10^5^–1.20 × 10^7^)	67 8.20 × 10^6^ (2.73 × 10^7^) 9.00 × 10^5^ (3.00 × 10^5^–4.00 × 10^6^)	0.002%
Gram-negative #	37 4.06 × 10^6^ (1.80 × 10^7^) 5.00 × 10^5^ (1.00 × 10^5^–8.00 × 10^5^)	32 2.42 × 10^7^ (1.11 × 10^8^) 1.00 × 10^6^ (3.20 × 10^5^–5.00 × 10^6^)	19 4.55 × 10^6^ (1.17 × 10^7^) 3.00 × 10^5^ (2.00 × 10^5^–2.00 × 10^6^)	0.083
*Veillonella* #	9 1.02 × 10^6^ (5.15 × 10^6^) 5.00 × 10^5^ (3.00 × 10^5^–9.00 × 10^5^)	6 8.12 × 10^6^ (1.52 × 10^6^) 1.45 × 10^6^ (6.00 × 10^5^–5.00 × 10^6^)	4 3.38 × 10^6^ (1.57 × 10^7^) 1.15 × 10^6^ (2.50 × 10^5^–6.50 × 10^6^)	0.42
*Neisseria* #	21 6.63 × 10^6^ (2.38 × 10^7^) 4.00 × 10^5^ (8.00 × 10^4^–2.00 × 10^6^)	15 4.59 × 10^7^ (1.62 × 10^8^) 9.00 × 10^5^ (3.00 × 10^5^–4.80 × 10^6^)	2 2.75 × 10^7^ (3.18 × 10^7^) 2.75 × 10^7^ (5.00 × 10^6^–5.00 × 10^7^)	0.14
*Staphylococci* #	9 2.04 × 10^5^ (1.94 × 10^5^) 1.00 × 10^5^ (5.00 × 10^4^–4.00 × 10^6^)	6 2.89 × 10^7^ (2.73 × 10^7^) 2.50 × 10^7^ (2.00 × 10^5^–4.00 × 10^6^)	9 2.47 × 10^6^ (2.63 × 10^6^) 1.70 × 10^6^ (4.00 × 10^5^–5.00 × 10^7^)	0.016 &
*Actinomyces* #	8 7.14 × 10^5^ (8.17 × 10^5^) 4.00 × 10^5^ (1.50 × 10^5^–1.30 × 10^6^)	9 1.57 × 10^6^ (1.11 × 10^6^) 1.00 × 10^6^ (1.00 × 10^6^–2.00 × 10^6^)	3 2.77 × 10^6^ (2.36 × 10^6^) 3.0 0 × 10^6^ (3.00 × 10^5^–5.00 × 10^6^)	0.2
*Streptococci* #	128 3.98 × 10^7^ (2.14 × 10^8^) 1.00 × 10^6^ (3.50 × 10^5^–3.00 × 10^6^)	95 3.30 × 10^7^ (1.17 × 10^8^) 3.00 × 10^6^ (8.00 × 10^5^–1.20 × 10^7^)	51 8.66 × 10^6^ (3.06 × 10^7^) 8.00 × 10^5^ (3.00 × 10^5^–3.00 × 10^6^)	0.034 &
*S. vetibularis*	20 1.71 × 10^7^ (4.57 × 10^7^) 1.30 × 10^6^ (4.50 × 10^5^–3.80 × 10^6^)	10 1.64 × 10^7^ (4.03 × 10^7^) 2.50 × 10^6^ (8.00 × 10^5^–3.00 × 10^6^)	3 2.97 × 10^6^ (3.52 × 10^6^) 1.40 × 10^6^ (5.00 × 10^5^–7.00 × 10^6^	0.38
*S. salivarius*	30 3.43 × 10^7^ (1.57 × 10^8^) 9.50 × 10^5^ (3.00 × 10^5^–2.00 × 10^6^)	10 7.25 × 10^6^ (1.27 × 10^7^) 3.00 × 10^6^ (8.00 × 10^5^–8.00 × 10^6^)	7 8.37 × 10^5^ (1.05 × 10^6^) 8.00 × 10^5^ (4.00 × 10^4^–1.00 × 10^6^)	0.22
*S. parapneumonie*	18 1.87 × 10^7^ (6.10 × 10^7^) 9.50 × 10^5^ (5.00 × 10^5^–2.20 × 10^6^)	7 5.83 × 10^6^ (5.28 × 10^6^) 5.00 × 10^6^ (1.40 × 10^6^–1.00 × 10^7^)	3 7.10 × 10^6^ (6.85 × 10^6^) 7.00 × 10^6^ (3.00 × 10^5^–1.4 0 × 10^7^)	0.72
*S. oralis*	26 1.28 × 10^7^ (4.36 × 10^8^) 1.20 × 10^6^ (7.00 × 10^5^–4.00 × 10^6^)	26 1.04 × 10^8^ (1.89 × 10^8^) 9.50 × 10^6^ (1.30 × 10^6^–4.00 × 10^7^)	17 6.93 × 10^7^ (4.83 × 10^7^) 6.00 × 10^5^ (5.00 × 10^5^–1.20 × 10^6^)	<0.001 *
*S. mitis*	19 1.42 × 10^7^ (5.00 × 10^7^) 1.10 × 10^6^ (2.00 × 10^5^–4.00 × 10^6^)	3 2.87 × 10^6^ (3.60 × 10^6^) 1.20 × 10^6^ (4.00 × 10^5^–7.00 × 10^6^)	9 4.79 × 10^6^ (7.61 × 10^6^) 9.00 × 10^5^ (3.00 × 10^5^–3.00 × 10^6^)	0.47
*Candida* spp.	9 7.00 × 10^2^ (6.52 × 10^2^) 5.00 × 10^2^ (2.00 × 10^2^–7.00 × 10^2^)	18 9.63 × 10^4^ (3.06 × 10^5^) 4.05 × 10^3^ (1.50 × 10^3^–1.8 × 10^4^)	0	0.015

Data are presented as mean (SD), median (Q1-Q3) or number. Buccal (A) and soft palate (B) mucosa, the tongue (C), palatal (Da) and buccal (Db) dental surface, gingival pocket (E). #—all identified strains; $—significant difference A + B vs. Da + Db. *p* = 0.001; %—significant difference A + B vs. Da + Db. *p* = 0.001; &—no significant differences in the post hoc analysis; *—significant difference Da + Db vs. E. *p* = 0.013.

## Data Availability

The data presented in this study are available on request from the corresponding author.

## Supplementary Materials

The following supporting information can be downloaded at: https://www.mdpi.com/article/10.3390/ijerph20032252/s1, Table S1. Microbiome diversity in sites A–E.
