# Supplementary material for: Oral Microbiota—One Habitat or Diverse Niches? A Pilot Study of Sampling and Identification of Oral Bacterial and Fungal Biota in Patients with Type I Diabetes Mellitus Treated with Insulin Pump
Source: Int J Environ Res Public Health. 2023 Jan 27;20(3):2252. doi: 10.3390/ijerph20032252 (PMC9914992; doi:10.3390/ijerph20032252)
Supplement: Supplementary file 1 [file ijerph-20-02252-s001.zip › ijerph-2125774-supplementary.pdf]

Table S1. Microbiome diversity in sites A–E.

Strain	Site						Number Sites with Strain (%)
	A	B	C	Da	Db	E	
Actinomyces (any)	X	X	X	X	X	X	6 (100%)
Actinomyces spp		X	X	X			3 (50%)
Actinomyces graevenitzii		X			X		2 (33.3%)
Actinomyces naeslundii				X			1 (16.7%)
Actinomyces odontolyticus	X	X	X		X	X	5 (83.3%)
Actinomyces oris					X	X	2 (33.3%)
Aerococcus viridans						X	1 (16.7%)
Alloscardovia spp			X				1 (16.7%)
Bacillus halosaccharovorans						X	1 (16.7%)
Bifidobacterium spp	X		X	X			3 (50%)
Bifidobacterium dentium	X						1 (16.7%)
Bifidobacterium longum			X				1 (16.7%)
Capnocytophaga granulosa					X		1 (16.7%)
Cutibacterium spp					X		1 (16.7%)
Cutibacterium acnes						X	1 (16.7%)
Eikenella spp	X						1 (16.7%)
Enterobacter cloacae	X						1 (16.7%)
Fusobacterium (any)				X	X	X	3 (50%)
Fusobacterium spp				X	X		2 (33.3%)
Fusobacterium canifelinum				X		X	2 (33.3%)
Fusobacterium nucleatum						X	1 (16.7%)
Gemella haemolysans	X	X					2 (33.3%)
Geotrichum spp			X				1 (16.7%)
Granulicatella adiacens		X					1 (16.7%)
Granulicatella elegans			X				1 (16.7%)
Hafnia alvei			X				1 (16.7%)
Klebsiella oxytoca	X					X	2 (33.3%)
Klebsiella pneumoniae	X						1 (16.7%)
Lactobacillus spp						X	1 (16.7%)
Legionella pneumophila			X				1 (16.7%)
Leptotrichia spp					X		1 (16.7%)
Micrococcus spp	X				X		2 (33.3%)
Micrococcus luteus						X	1 (16.7%)
Neisseria (any)	X	X	X	X	X	X	6 (100%)
Neisseria spp				X	X		2 (33.3%)
Neisseria flavescens					X		1 (16.7%)
Neisseria macacae		X					1 (16.7%)
Neisseria perflava			X		X		2 (33.3%)
Neisseria subflava	X	X	X	X	X	X	6 (100%)
Prevotella (any)		X	X	X		X	4 (66.7%)
Prevotella spp				X			1 (16.7%)
Prevotella buccae						X	1 (16.7%)
Prevotella denticola						X	1 (16.7%)
Prevotella maculosa						X	1 (16.7%)
Prevotella nigrescens						X	1 (16.7%)
Prevotella oralis						X	1 (16.7%)
Prevotella oris		X				X	2 (33.3%)

Prevotella pallens			X				1 (16.7%)
Prevotella salivae		X					1 (16.7%)
Raoultella ornithinolytica				X			1 (16.7%)
Rothia dentocariosa				X	X		2 (33.3%)
Rothia terrae		X					1 (16.7%)
Selenomonas spp						X	1 (16.7%)
Selenomonas flueggei				X			1 (16.7%)
Selenomonas infelix						X	1 (16.7%)
Serratia urealytica				X			1 (16.7%)
Sporosarcina luteola	X						1 (16.7%)
Staphylococcus (any)	X	X	X	X	X	X	6 (100%)
Staphylococcus spp			X	X			2 (33.3%)
Staphyloccus warneri						X	1 (16.7%)
Staphylococcus aureus	X		X		X	X	4 (66.7%)
Staphylococcus epidermidis	X					X	2 (33.3%)
Staphylococcus haemolyticus		X				X	2 (33.3%)
Staphylococcus hominis		X	X	X			3 (50%)
Staphylococcus pasteuri			X				1 (16.7%)
Staphylococcus warneri	X	X			X	X	4 (66.7%)
Streptococcus (any)	X	X	X	X	X	X	6 (100%)
Streptococcus spp	X	X	X	X	X	X	6 (100%)
Straptococcus salivarius		X					1 (16.7%)
Streprococcus anginosus					X		1 (16.7%)
Streptococcus anginosus	X			X	X	X	4 (66.7%)
Streptococcus cristatus				X	X		2 (33.3%)
Streptococcus gordonii	X			X	X		3 (50%)
Streptococcus infantis			X			X	2 (33.3%)
Streptococcus intermedius				X	X		2 (33.3%)
Streptococcus mitis	X	X	X	X	X	X	6 (100%)
Streptococcus mutans				X	X		2 (33.3%)
Streptococcus oralis	X	X	X	X	X	X	6 (100%)
Streptococcus parasangiunis		X					1 (16.7%)
Streptococcus parasanguinis	X	X	X	X	X	X	6 (100%)
Streptococcus pneumoniae	X	X	X	X	X	X	6 (100%)
Streptococcus salivarius	X	X	X	X	X	X	6 (100%)
Streptococcus sanguinis		X		X	X	X	4 (66.7%)
Streptococcus sorbinus	X			X	X		3 (50%)
Streptococcus vestibularis	X	X	X	X	X	X	6 (100%)
Turicella spp						X	1 (16.7%)
Veillonella (any)	X	X	X	X	X	X	6 (100%)
Veillonella spp			X				1 (16.7%)
Veillonella atypica	X	X	X			X	4 (66.7%)
Veillonella dispar	X						1 (16.7%)
Veillonella parvula	X		X	X	X	X	5 (83.3%)
Candida (any)	X	X	X	X	X		5 (83.3%)
Candida spp				X	X		2 (33.3%)
Candida albicans	X	X	X	X	X		5 (83.3%)
Candida dubliniensis				X	X		2 (33.3%)
